# Brain Medical Image Fusion Based on Dual-Branch CNNs in NSST Domain

**DOI:** 10.1155/2020/6265708

**Published:** 2020-04-14

**Authors:** Zhaisheng Ding, Dongming Zhou, Rencan Nie, Ruichao Hou, Yanyu Liu

**Affiliations:** School of Information, Yunnan University, Kunming 650504, China

## Abstract

Computed tomography (CT) images show structural features, while magnetic resonance imaging (MRI) images represent brain tissue anatomy but do not contain any functional information. How to effectively combine the images of the two modes has become a research challenge. In this paper, a new framework for medical image fusion is proposed which combines convolutional neural networks (CNNs) and non-subsampled shearlet transform (NSST) to simultaneously cover the advantages of them both. This method effectively retains the functional information of the CT image and reduces the loss of brain structure information and spatial distortion of the MRI image. In our fusion framework, the initial weights integrate the pixel activity information from two source images that is generated by a dual-branch convolutional network and is decomposed by NSST. Firstly, the NSST is performed on the source images and the initial weights to obtain their low-frequency and high-frequency coefficients. Then, the first component of the low-frequency coefficients is fused by a novel fusion strategy, which simultaneously copes with two key issues in the fusion processing which are named energy conservation and detail extraction. The second component of the low-frequency coefficients is fused by the strategy that is designed according to the spatial frequency of the weight map. Moreover, the high-frequency coefficients are fused by the high-frequency components of the initial weight. Finally, the final image is reconstructed by the inverse NSST. The effectiveness of the proposed method is verified using pairs of multimodality images, and the sufficient experiments indicate that our method performs well especially for medical image fusion.

## 1. Introduction

In recent decades, image fusion has played an essential role in the field of image processing [1]. It is a kind of image enhancement technology whose purpose is to generate an informative image by fusing two or more images under the same scene from various sensors that contain complementary information. It is quite obvious that the final image inherits significant information from all the source images. Nowadays, image fusion technique has been further developed in many fields and widely employed in medical applications [2].

Medical imaging takes many forms and is classified according to structure and functional information into positron emission computed tomography (PET), computed tomography (CT), and magnetic resonance imaging (MRI) [3]. Medical image fusion is to fuse complementary information from the different modal sensors to enhance the visual perception [4].

Recently, the methods based on multiscale transform (MST) are a widely discussed transform theory in image processing. The multiscale transform tools include Laplacian pyramid (LAP) [5], ratio of low-pass pyramid (RP) [6], dual-tree complex wavelet transform (DTCWT) [7], contourlet transform (CT) [8], and non-subsampled contourlet transform (NSCT) [9]. Those fusion methods all consist of three steps: decomposition, fusion, and reconstruction. By comparing those methods, it is evident that NSCT generally achieves more information from the source images to achieve the best results. The fundamental reason is that the NSCT method not only has the characteristic of multiresolution and time-frequency local of wavelet transform but also has multidirectivity and anisotropy. However, the operating efficiency of the NSCT method is time-consuming. In view of that, the non-subsampled shearlet transform (NSST) method is created to greatly improve the utilization rate of resources [10]. Of course, in the process of the subfields of information processing, not only the decomposition methods, the fusion strategies also play an important role. Conventionally, the high-frequency band fusion strategies are selected in many ways, while low-frequency bands usually choose the average weight coefficient as the fusion strategy. According to the researches, one of the most crucial issues is to calculate the weight maps from the source images [11]. In addition, in most MST-based fusion methods, the low-frequency bands have achieved less attention. However, the kind of activity measurement and weight assignment are not in all cases on account of many factors such as noise, misregistration, and the difference between source pixel intensities [12]. Furthermore, many recently existed methods had made many changes in the fusion methods and elaborated on weight assignment strategies [13]. Unfortunately, it is actually a difficult task to design an ideal activity level measurement or weight assignment strategy to comprehensively take all the key issues of fusion into account.

Nowadays, deep learning gets increasing attention in the field of computer vision perception, because the deep learning network architecture has the following two advantages [14]. On the one hand, because the artificial neural network has multiple hidden layers, it is obviously better than many traditional neural networks in feature learning ability. On the other hand, the difficulty of training deep neural network is reduced by implementing layer-wise pretraining through an unsupervised learning method. Deep learning simulates the hierarchical structure of visual perception system which makes deep learning have excellent performance in presentation and learning. Convolutional neural networks (CNNs) are a typical deep learning model [15]; Li et al. introduced a fusion method combining a Dual-Channel Spiking Cortical Model (DCSCM) and CNNs [12]. It introduces the CNNs to encode a direct mapping from the source image to weight map, which is the fusion framework of the low-frequency coefficients. Liu et al. proposed a multifocus image fusion method with CNNs, which mentioned CNNs to extract the focus region and acquire a decision weight map. It has been proved that the results fused by CNNs are better than those from the traditional shallow neural network. What is more, CNNs consider the nonlinear features of images, while traditional pixel level methods fail to get high level features. It can effectively filter redundant information through convolution and pooling layer [16].

However, there are still shortcomings in the above methods. Many MST-based methods are perfectly unsuitable for medical image fusion; for example, the RP method is usually used for the fusion of infrared visible, but the artifacts will be generated when it is applied on medical image. Due to the large difference of the same part in a group of medical images, if the weight acquired by CNNs is directly introduced for fusing the original images, a lot of information will be lost [17]. Fortunately, the NSST method solves the defect of information loss in the sampling step by decomposing the image into directional subbands at different scales and obtains the multiangle information of the image accurately at the same time. The NSST method has many advantages that other sparse decomposition methods do not have [11, 18, 19]. Based on the idea of NSCT, NSST improved the method to achieve higher operating efficiency than NSCT, and at the same time, it was able to obtain more sparse decomposition results than methods curvelet, contourlet, and wavelet [20]. The application of the NSST algorithm in medical images will not generate artifacts and even retain the specific soft tissue and bone structure information in medical images. At the same time, we bring in CNNs to overcome difficulties named designing robust activity level measurement and weight allocation strategies. In fact, CNNs directly map the source image to the weight map after training [21]. By this, some issues are jointly resolved by learning network parameters in an “optimal” manner. In addition, to address the problem that the initial weight is inapplicable to medical images, the initial weight is represented in multiscale domain as the high- and low-frequency coefficients.

In this paper, we commence to deploy a fusion framework that combines CNNs and NSST which simultaneously contains the advantages of them both. Firstly, the source images {*A*, *B*} are decomposed by NSST to get their low-frequency coefficients {*L*_*A*_, *L*_*B*_} and high-frequency coefficients {*H*_*A*_^*l*,*k*^, *H*_*B*_^*l*,*k*^}. Moreover, the weight *W*_*S*_ is also decomposed by NSST to multiple scales {*W*_*S*_^*L*^, *W*_*S*_^*H*,*k*^}. Then, the high-frequency component of the weight *W*_*S*_^*H*,*k*^ is used for high-frequency coefficient {*H*_*A*_^*l*,*k*^, *H*_*B*_^*l*,*k*^} fusion to obtain the fused high-frequency fusion coefficients *H*_*F*_^*l*,*k*^. The low-frequency coefficients are divided into two parts, a part coefficient {*L*_*A*1_, *L*_*B*1_} which is the first component of the low-frequency coefficients by a novel strategy which avoids both energy conservation and detail extraction problems. The other part {*L*_*A*2_, *L*_*B*2_} which is the second component of the low-frequency coefficients named low2 is fused by the spatial frequency of low-frequency component of the weight *W*_*S*_^*L*^. At last, the final image is reconstructed by the inverse NSST. The effectiveness of this method is verified with pairs of multimodality brain image fusion, and the results of the experiments indicate that the proposed fusion method performs well, especially for the fusion of medical images.

The rest of this paper is structured in the following fashion.  Section 2 presents the whole fusion framework and analyzes the subpart in detail.  Section 3 shows the detailed fusion strategies. Experimental results and analysis are summarized in Section 4. The conclusions are given in Section 5.

## 2. Theoretical Basis

### 2.1. Non-subsampled Shearlet Transform

NSST, which was referenced in [10], is conducive to better maintaining the edge information and contour structure of images. NSST uses the nonsampling pyramid transformation (NSP) and the shearlet filter (SF) to achieve shift invariance which makes up for the shortcomings of the contourlet transform (CT). NSP is a multiscale analysis of the NSST with translation invariant filter structure, which goes for the same multiscale analysis characteristics as LP decomposition. The equivalent filters of the *k*th level cascading NSP are as follows:

(1) $$\begin{array}{c} H_{n}^{\text{eq}}\left(z\right)=\left\{\begin{array}{c} H_{1}\left(z^{2^{n-1}}\right)\overset{n-2}{\underset{j=0}{\prod }}H_{0}\left(z^{2^{j}}\right),\;1\leq n<2^{k},\\ \overset{n-1}{\underset{j=0}{\prod }}H_{0}\left(z^{2^{j}}\right),\;n=2^{k}, \end{array}\right. \end{array}$$

where *z*^*j*^ stands for [*z*_1_^*j*^, *z*_2_^*j*^].

Shearlet transform is a sparse representation method of nearly optimal multidimensional functions according to the synthetic expansion affine system, as shown in equation (3). When

(2) $$\begin{array}{c} X=\left[\begin{array}{cc} 4 & 0\\ 0 & 2 \end{array}\right],\\ Y=\left[\begin{array}{cc} 1 & 1\\ 0 & 1 \end{array}\right], \end{array}$$

the synthetic wavelets turn into shearlet.

(3) $$\begin{array}{c} \Lambda_{AB}\left(\psi \right)=\left\{\psi_{j,l,k}\left(x\right)={\left|\det X\right|}^{j/2}\psi \left(Y^{l}X^{j}x-k\right)\right\}, \end{array}$$

where *j*, *l* ∈ **Z**, *k* ∈ **Z**^2^.

**Figure 1:**
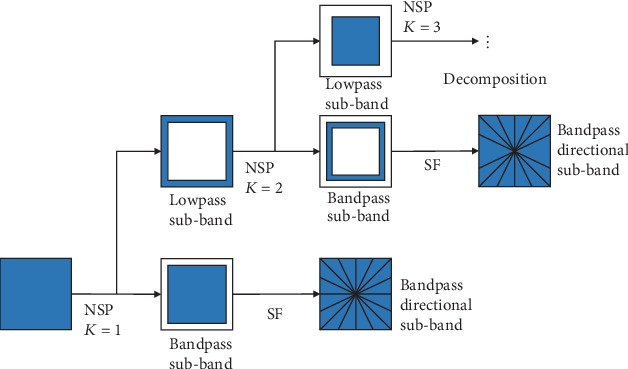
NSST decomposition block diagram.

The NSST is to combine the 2D NSP and the SF, and the result of the filtering structure is equal to the ideal partition of the frequency plane. The NSST decomposition block diagram is shown in Figure 1.

### 2.2. Convolutional Neural Networks

The idea of CNN was first proposed by LeCun in 1989 which has been successfully applied in the recognition of English handwriting, and a CNN-based method performed exceedingly good results which were demonstrated in [22]. CNN consists of input and output layers and multiple hidden layers, which are divided into convolutional layer, pooling layer, and fully connected layer. The input layer mainly preprocesses the original image. The convolution layer which is the most important layer of CNN includes two key operations, namely, local associations and sliding window. The convolution layer is the feature extraction layer, and the calculation process is as follows:

(4) $$\begin{array}{c} a_{j}^{n}=\sigma \left(\underset{i\in M_{j}^{n}}{\sum }a_{i}^{n-1}*k_{ij}^{n}+b_{j}^{n}\right), \end{array}$$

where *a*_*j*_^*n*^ is the calculation results of the *j*th node in the *n*th layer, *M*_*j*_^*n*^ is the index set of multiple input feature graphs corresponding to the *j*th output feature graph in the *n*th layer, *b*_*j*_^*n*^ is a common bias term of all input feature graphs, and *k*_*ij*_^*n*^ is the convolution kernel.

The pooling layer is sandwiched between successive convolution layers and is mainly helpful for image compression. Both of the reducing feature dimension and preventing overfitting are carried out through the pooling layer operation. The calculation process of the pooling layer is as follows:

(5) $$\begin{array}{c} a_{j}^{n}=\sigma \left(\beta_{j}^{n}\text{down}\left(a_{j}^{n-1}\right)+b_{j}^{n}\right), \end{array}$$

where the function down(·) is a downsampling function and *β* is a specific multiplicative bias to correspond to the output of the function.

The output layer is fully connected which fully excavates the mapping relationship between the features extracted at the end of the network and the output category tags.

**Figure 2:**
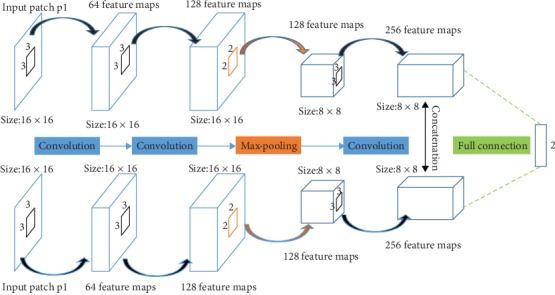
The architecture of the dual-branch network for training.

The convolutional network introduced in our fusion strategy is shown in Figure 2, which is a Siamese network which shares the same architecture and weights around the two branches [21, 23]. Each of the branch contains three convolutional layers and a max-pooling layer. The feature maps of two branches are concatenated and then pass through a fully connected layer which is viewed as the weight assignment part of a pair of the fusion method. The input images {*A*, *B*} are subject to a 2-dimensional vector through the dual-branch network and then through a Softmax layer to produce a probability distribution over two classes {0 or 1}. Finally, a weight map *S* is finally achieved by assigning the value of all the pixels within the location and averaging the overlapped pixels. We make use of high-quality image patches and their blurred version to train the network. The training process is operated on the popular deep learning framework Caffe [24], and there is a detailed training process in [23]. Moreover, the work has demonstrated the extraordinary suitability of the CNNs for image fusion. On account of that, we introduce the network architectures as the feature extractor directly and remove a full-connection layer to gain time.

## 3. Fusion Strategies

**Figure 3:**
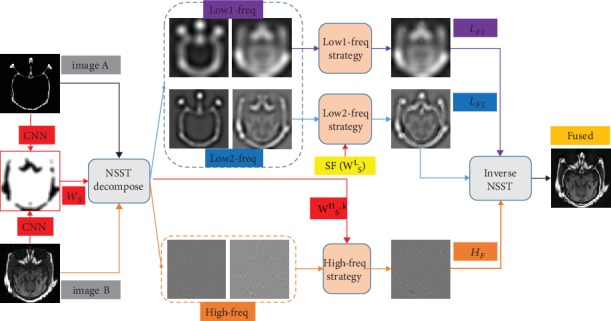
The schematic diagram of our fusion framework.

First of all, the overview of the proposed brain medical image fusion framework is shown in Figure 3. Each part of the fusion framework will be analyzed in detail, and the advantages will be exhibited in this section. In particular, the initial weights taken out by CNN is also decomposed by NSST to get the low- and high-frequency components of the weights. Spatial frequency of low-frequency component is setting as the fusion strategy of the low2-frequency coefficient.

### 3.1. Low-Frequency Coefficient Fusion Strategy

Generally, an image is regarded as a two-dimensional piecewise smooth signal [25], and most of its energy is commonly contained in the low-frequency coefficients. Furthermore, the image edge and contour information are contained in the high-frequency coefficients. In MST-based fusion, the choice of low-frequency fusion strategy also affects the final fusion result. The simple weighted averaging and maximum-based strategies are the most common fusion strategies. When the low-frequency coefficients are fused, those fusion strategies tend to lose the energy of the images, resulting in poor fusion effect. Indeed, the brightness of some areas may drop sharply, resulting in decreased visual perception. To tackle the above issues, this paper introduces WLE_s_ which is an activity level measure.

(6) $$\begin{array}{c} {\text{WLE}}_{\text{s}}\left(u,v\right)=\overset{R}{\underset{i=-R}{\sum }}\overset{R}{\underset{j=-R}{\sum }}W\times \left(i+R+1,j+R+1\right)L_{S}{\left(u+i,v+j\right)}^{2}, \end{array}$$

where *S* ∈ (*A*, *B*), *A* and *B* are the source images, and *W* is a (2*R* + 1) × (2*R* + 1) weighting matrix with radius *R*. The value of each element in *W* is set to 2^2*R*−*r*^, where *r* is the distance of its four-neighborhood to the center.

It is known to us all that NSST decomposition has some limitations because of some factors, for example, computational efficiency. As a result, to improve the ability of WLE_s_ in detail extraction, the weighted sum of WSEML_s_ is defined as

(7) $$\begin{array}{c} {\text{WSEML}}_{\text{s}}\left(u,v\right)=\overset{R}{\underset{i=-R}{\sum }}\overset{R}{\underset{j=-R}{\sum }}W\left(i+R+1,j+R+1\right)\times {\text{EML}}_{\text{s}}\left(u+i,v+j\right), \end{array}$$

where EML_s_ is as follows:

(8) $$\begin{array}{c} {\text{EML}}_{\text{s}}\left(u,v\right)=\left|2S\left(u,v\right)-S\left(u-1,v\right)-S\left(u+1,v\right)\right|+\left|2S\left(u,v\right)-S\left(u,v-1\right)-S\left(u,v+1\right)\right|+\frac{1}{\sqrt{2}}\left|2S\left(u,v\right)-S\left(u-1,v-1\right)-S\left(u+1,v+1\right)\right|+\frac{1}{\sqrt{2}}\left|2S\left(u,v\right)-S\left(u-1,v+1\right)-S\left(u+1,v-1\right)\right|. \end{array}$$

The multiplication of WLE and WSEML is defined as the final activity level measure, and the first components of the low-frequency coefficients are defined as {*L*_*A*1_, *L*_*B*1_}. The fusion of this part is calculated according to

(9) $$\begin{array}{c} L_{F1}\left(u,v\right)=\left\{\begin{array}{c} L_{A1}\left(u,v\right),\;\text{if WL}{\text{E}}_{A}\left(u,v\right)\times {\text{WSEML}}_{A}\left(u,v\right)\geq {\text{WLE}}_{B}\left(u,v\right)\times {\text{WSEML}}_{B}\left(u,v\right),\\ L_{B1}\left(u,v\right),\;\text{otherwise}. \end{array}\right. \end{array}$$

The other part of low-frequency coefficient fusion strategy uses the CNN-based weight map to achieve the final coefficients. Feed the source images *A* and *B* to the branches of the convolutional network and obtain the saliency map *W*_*S*_. Then, we calculate the spatial frequency of *W*_*S*_^*L*^ which is the low-frequency component of *W*_*S*_ that can be taken from NSST as the weight of fusion strategy. The process formula is shown in equation (10). The part coefficients are defined as {*L*_*A*2_, *L*_*B*2_}, and the calculation of the fusion process is as follows:

(10) $$\begin{array}{c} \left\{\begin{array}{c} W_{S}^{SF,L}=SF\left(W_{S}^{L}\right),\\ SF\left(W_{S}^{L}\right)=\sqrt{{RF}^{2}+{CF}^{2}},\\ RF=\sqrt{\frac{1}{mn}\overset{M-1}{\underset{i=1}{\sum }}\overset{N-1}{\underset{j=1}{\sum }}{\left(W_{S}^{L}\left(i,j\right)-W_{S}^{L}\left(i,j+1\right)\right)}^{2}},\\ CF=\sqrt{\frac{1}{mn}\overset{M-1}{\underset{i=1}{\sum }}\overset{N-1}{\underset{j=1}{\sum }}{\left(W_{S}^{L}\left(i,j\right)-W_{S}^{L}\left(i+1,j\right)\right)}^{2}}, \end{array}\right. \end{array}$$

(11) $$\begin{array}{c} L_{F2}=L_{A2}\times W_{S}^{SF,L}+L_{B2}\times \left(1-W_{S}^{SF,L}\right). \end{array}$$

### 3.2. High-Frequency Coefficient Fusion Strategy

**Figure 4:**
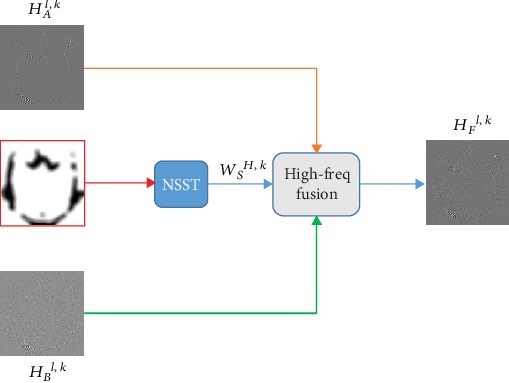
The schematic diagram of high-frequency coefficient fusion strategy.

At present, researches on high-frequency coefficient fusion strategies are in-depth, including various methods such as regional energy, Pulse-Coupled Neural Network (PCNN), and sparse fusion [26]; however, these strategies have some drawbacks in extraction of detail information. It is known to us that CNN has absolute advantages to extract detail information from the source image. Therefore, this section regards the weight map *W*_*S*_ extracted from the source images by CNN as the key for high-frequency coefficient fusion. The high-frequency coefficients of the source image {*A*, *B*} are defined as {*H*_*A*_^*l*,*k*^, *H*_*B*_^*l*,*k*^}. The schematic diagram of high-frequency coefficient fusion strategy is shown in Figure 4. The calculation of the high-frequency fusion is as follows:

(12) $$\begin{array}{c} H_{F}^{l,k}=W_{S}^{H,k}\times H_{A}^{l,k}+\left(1-W_{S}^{H,k}\right)\times H_{B}^{l,k}. \end{array}$$

### 3.3. Detailed Fusion Scheme and Analysis

**Figure 5:**
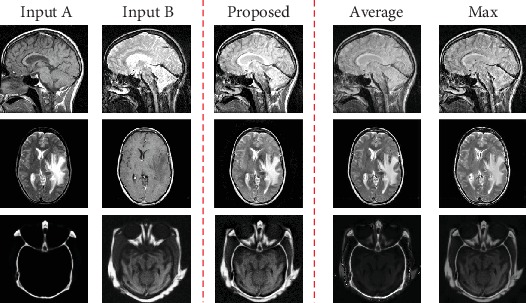
NSST-based fusion strategy compared.

In this fusion scheme, we just only consider the fusion of two source images. The detailed fusion scheme is described in the following steps. In order to effectively analyze the results, we analyzed introducing the same decomposition method which means we only use the NSST-based method but different fusion strategy choices to fuse the images. As shown in Figure 5, the results by the simple weighted averaging or maximum value-based strategies do not consider the relationship between pixels, resulting in the overall brightness and contrast of the image which are slightly worse. By contrast, the results obtained by the proposed method in this paper retain more abundant information and the details are clear. The contour information in the low-frequency image is retained completely through our low-frequency strategy, while the contrast and brightness information are retained by the high-frequency strategy. In addition, we found that through the feature screening of CNNs, the important features of the original images were basically retained, such as bone structure in CT images and soft tissue vessels in MRI images. Therefore, it is reasonable to believe that the proposed low- and high-frequency fusion strategies are more effective than the average and maximum strategies.

**Step 1 .**

Decompose the source images {*A*, *B*} by using NSST to attain their low-frequency coefficients {*L*_*A*1_, *L*_*B*1_} and {*L*_*A*2_, *L*_*B*2_} and the high-frequency coefficients {*H*_*A*_^*l*,*k*^, *H*_*B*_^*l*,*k*^} at each *K* scale and *l* direction.

**Step 2 .**

Feed the source images to CNN to acquire the weight map *W*_*S*_. Decompose the weight map by NSST to low- and high-frequency coefficients {*W*_*S*_^*L*^, *W*_*S*_^*H*,*k*^}.

**Step 3 .**

Fuse low-frequency coefficients by the algorithm in Section 3.1 and receive the fusion coefficient {*L*_*F*1_, *L*_*F*2_}.

**Step 4 .**

Use the method in Section 3.2 to fuse the high-frequency coefficients and obtain the high-frequency fusion coefficients *H*_*F*_^*l*,*k*^.

**Step 5 .**

Perform inverse NSST on {*L*_*F*1_, *L*_*F*2_, *H*_*F*_^*l*,*k*^} to reconstruct the final image *F*.

## 4. Experiments

### 4.1. Experimental Settings

**Figure 6:**
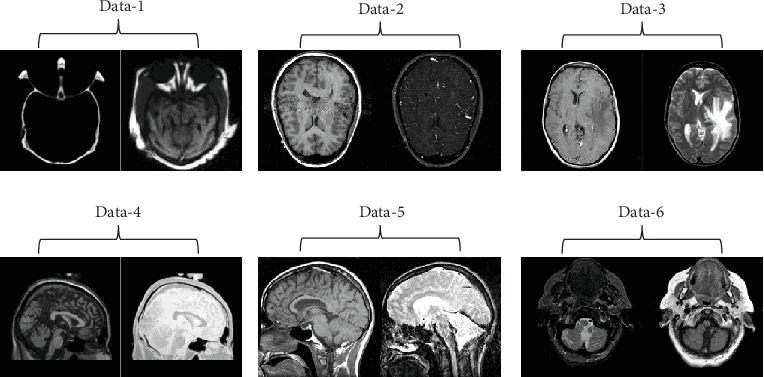
Source images in the experiments.

The simulation experiments were carried out by MATLAB2018a software on PC with Intel i7 7700 3.6 GHz, 24 GB RAM. Several experiments have been performed to analyze the effects of the proposed method. All of the images are 256 × 256 grayscale images. Each pair of the source images has been accurately registered which could be collected from http://www.med.harvard.edu/AANLIB/. The source images are presented in Figure 6.

### 4.2. Comparison Methods

We compared our method with seven representative methods: LP method [27], DTCWT [28], curvelet transform (CVT) method [29], sparse representation with CVT (SR-CVT) method [18], NSCT-PCNN-based [30], NSST-SR [31], and NSST-PAPCNN [11]. Among them, LP and DTCWT methods are the classical algorithms. In particular, the LP method has superior performance in medical image fusion. NSST-SR and NSST-PAPCNN are both outstanding MST-based fusion strategies. What is more, NSST-PAPCNN was just recently initiated within one year. Other contrast methods are often considered the contrast goals in the past few years. To show the difference between the experimental results intuitively, we mark the obvious difference area on a red rectangle. So, the contrast of the results of comparison methods is observed easily. The detailed result analysis is carried out in Section 4.4.

### 4.3. Quantitative Comparison

The subjective evaluation only involves the qualitative evaluation made by human, which takes human as the observer to make subjective qualitative evaluation on the advantages and disadvantages of the image. The selection of observers is generally considered to be untrained “amateurs” or trained “experts.” This method is based on statistical significance. In order to ensure that the subjective evaluation of the image is statistically significant, enough observers should participate in the evaluation. Because of this, human judgment is highly subjective and cannot guarantee the judgment. Objective evaluation is usually evaluated by testing the performance of multiple factors that affect image quality and calculating the consistency between quantized image quality and human subjective observation. It is another performance evaluation of fusion results besides subjective visual index. The combination of both evaluations makes the judgment of result more accurate. Usually, multiple objective metrics are applied to evaluate the performance of the fusion results comprehensively. Six widely recognized objective fusion metrics are presented as follows in brief. Those objective quantitative evaluation metrics include mutual information (MI) [32], mean structural similarity (MSSIM) [33], standard deviation (SD) [34], edge intensity (EI) [35], average gradient (AG) [36], and nonlinear correlation information entropy (*Q*_ncie_) [37].

1. MI measures the degree of the correlation between the two sets of data. The larger the value of MI, the richer the pixel grayscale and the more even the grayscale distribution. MI is defined as follows:

(13) $$\begin{array}{c} \text{MI}\left(R,F\right)=\overset{L}{\underset{u=1}{\sum }}\overset{L}{\underset{v=1}{\sum }}h_{R,F}\left(u,v\right)\log_{2}\frac{h_{R,F}\left(u,v\right)}{h_{R}\left(u\right)h_{F}\left(v\right)}, \end{array}$$

where *L* is the number of the gray level, *h*_*R*,*F*_(*u*, *v*) is the gray level histogram, besides, *h*_*R*_(*u*) and *h*_*F*_(*v*) are the edge histogram of the image *R* and *F*, *R* is the input image such as *A* or *B*, and MI of fused image can be represented by the following formula:

(14) $$\begin{array}{c} \text{MI}\left(A,B,F\right)=\text{MI}\left(A,F\right)+\text{MI}\left(B,F\right), \end{array}$$

in which MI(*A*, *B*, *F*) shows the total amount of information

(2) SSIM is an effective measure of correlation of the images, which is defined as the following formula:

(15) $$\begin{array}{c} \text{SSIM}\left(u,v\right)=\frac{\left(2\mu_{u}\mu_{v}+C_{1}\right)\left(2\sigma_{uv}+C_{2}\right)}{\left({\mu_{u}}^{2}+{\mu_{v}}^{2}+C_{1}\right)\left({\sigma_{u}}^{2}+{\sigma_{v}}^{2}+C_{2}\right)}, \end{array}$$

where *μ*_*u*_, *σ*_*u*_, and *σ*_*uv*_ indicate the mean, standard deviation, and crosscorrelation, respectively, and *C*_1_ and *C*_2_ are both constant. The value of MSSIM is derived by calculating the SSIM of images *A* and *B* with image *F*. The calculation equation of MSSIM is

(16) $$\begin{array}{c} \text{MSSIM}=\frac{\text{SSIM}\left(A,F\right)+\text{SSIM}\left(B,F\right)}{2}. \end{array}$$

The larger the value of MSSIM, the more similar the structure information is between the original images, which means the quality of result is better

(3) SD is a measure of how widely a set of values is dispersed from the mean. The calculation of SD of the final image is defined as follows:

(17) $$\begin{array}{c} \text{SD}=\sqrt{\frac{1}{M\times N}\overset{M}{\underset{u=1}{\sum }}\overset{N}{\underset{v=1}{\sum }}{\left(F\left(u,v\right)-\mu \right)}^{2}}, \end{array}$$

where *μ* is the mean value and *M* × *N* is the pixel of the ultimate image. A large standard deviation represents a large difference between most values and their mean. When SD is used as an objective evaluation metric, the larger the value of SD means that the contrast of the image is greater

(4) EI is essentially the amplitude of edge point gradient. The larger the value of EI, the richer the edge information of the image. Take the gradient value of each pixel of the final image *F*(*u*, *v*). The calculation of EI is defined as the follows:

(18) $$\begin{array}{c} \text{EI}\left(u,v\right)=\sqrt{\nabla xF{\left(u,v\right)}^{2}+\nabla yF{\left(u,v\right)}^{2}}, \end{array}$$

where ∇*xF*(*u*, *v*) and ∇*yF*(*u*, *v*) are the first differences of image *F* in the *x* and *y* directions of row *u* and column *v*. The equation of ∇*xF*(*u*, *v*) and ∇*yF*(*u*, *v*) is

(19) $$\begin{array}{c} \left\{\begin{array}{c} \nabla xF\left(u,v\right)=F\left(u,v\right)-F\left(u-1,v\right),\\ \nabla yF\left(u,v\right)=F\left(u,v\right)-F\left(u,v-1\right) \end{array}\right. \end{array}$$

(5) AG is the definition of the image which reflects the ability of the image to compare details. The greater the AG is, the more layers the image will have and the clearer it will be. AG is defined as

(20) $$\begin{array}{c} \text{AG}=\frac{1}{M\times N}\overset{M}{\underset{u=1}{\sum }}\overset{N}{\underset{v=1}{\sum }}\sqrt{\frac{{\left(\partial f/\partial x\right)}^{2}+{\left(\partial f/\partial y\right)}^{2}}{2}}, \end{array}$$

where *∂f*/*∂x* and *∂f*/*∂y* are the gradients in the horizontal and vertical directions

*Q*_ncie_ is a new nonlinear correlation information entropy for multivariable analysis which effectively judges the capacity of retaining the nonlinear information of the image. *Q*_ncie_ is represented by the following formula:

(21) $$\begin{array}{c} Q_{\text{ncie}}\left(X,Y\right)=2+\overset{b^{2}}{\underset{i=1}{\sum }}\frac{n_{i}}{N}\log_{b}\left(\frac{n_{i}}{N}\right), \end{array}$$

where *N* is the size of the dataset, *n*_*i*_ is the number of samples distributed in the *i*th rank grid, and *b* is set to $\sqrt{N}$, (1 ≤ *i* ≤ *K*, 1 ≤ *j* ≤ *K*)

The adopted metrics represent the quality of the image. In order to achieve better results in all aspects of fusion effect, the adopted six metrics all require larger values, but the maximum value of SSIM is 1. The quality metrics of the results of the objective quantitative assessments are shown in Table 1. In all fusion results, the best results are marked by bold.

### 4.4. Experimental Results and Analysis

**Figure 7:**
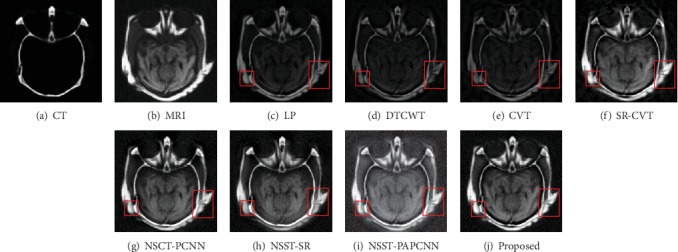
Fusion results of different methods in “Data-1”.

**Figure 8:**
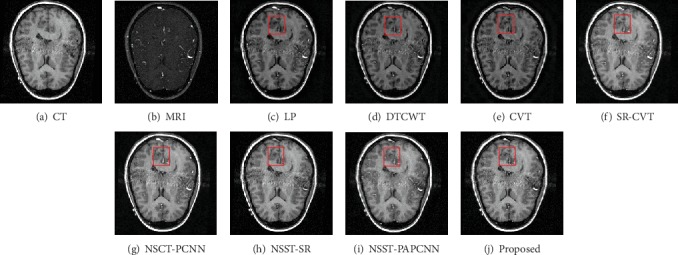
Fusion results of different methods in “Data-2”.

**Figure 9:**
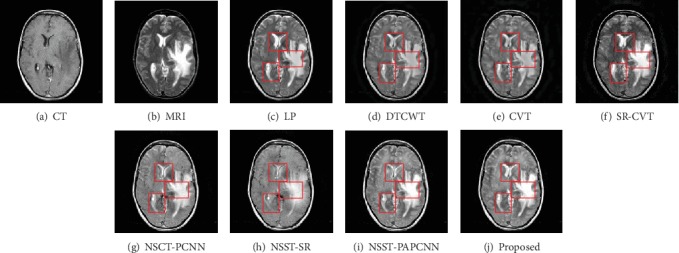
Fusion results of different methods in “Data-3”.

**Figure 10:**
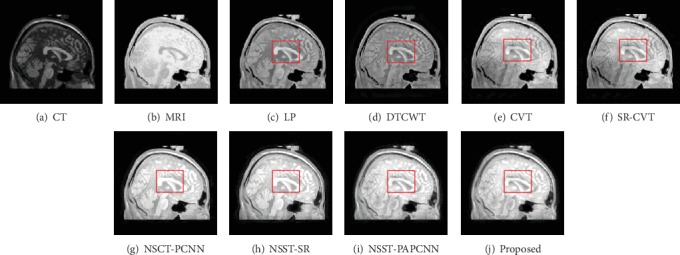
Fusion results of different methods in “Data-4”.

**Figure 11:**
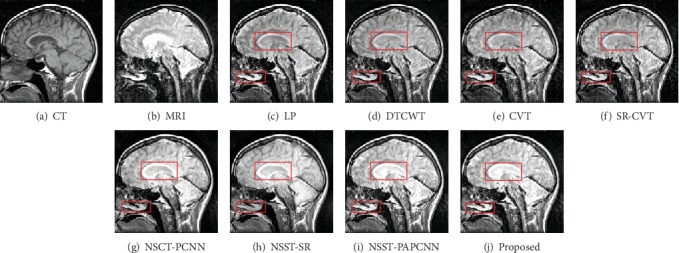
Fusion results of different methods in “Data-5”.

**Figure 12:**
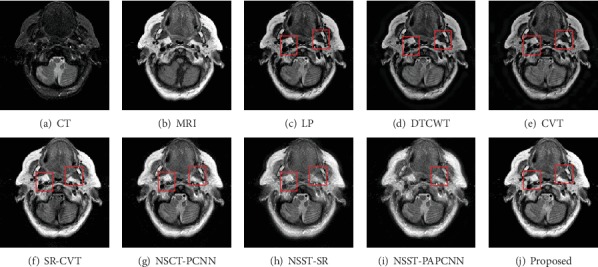
Fusion results of different methods in “Data-6”.

In this section, we show the results of our fusion method and the comparison experiments from Figures 7–12. What is more, we conduct subjective and objective analyses according to the results and the value of evaluation indicators.

Experimental results indicate that the designed fusion method has excellent performance in both detail information extraction and image energy retention. The results of different fusion methods for “Data-1” image set are shown in Figure 7. The CT and MRI images are shown in Figures 7(a) and 7(b), respectively. And then, Figures 7(c)–7(j) represent the results of the fusion methods such as LP, DTCWT, CVT, SR-CVT, NSCT-PCNN, NSST-SR, NSST-PAPCNN, and the proposed method.

Generally, the brain medical image fusion technology requires high accuracy and stability. Unfortunately, the different fusion methods have slightly different performance in contrast and detail preservation. To highlight the differences between the results of comparison methods, we mark the experimental results with red rectangle. As shown in Figures 7(f)–7(j), the color of the fused images is brighter than the other three comparison results. The results using NSCT-PCNN, NSST-SR, and NSST-PAPCNN shown in Figures 7(g)–7(i) preserved more bone structures of the CT image, but they missed soft tissues of the MRI image compared with our method. We observe visually in Figure 7(j) that either of the two red rectangles contains the most information than others. The same is true for the results in Figure 8. As shown in Figure 8, however, the result is different from the first two. The results using LP and SR-CVT preserve more details of the MRI image, but they do not hold back the spatial resolution of the CT image. Besides, the results of DTCWT and CVT both lose more contrast information.

The result of “Data-4” is shown in Figure 10. The result of the proposed method has almost a better visual effect than others. The DTCWT, CVT, NSCT-PCNN, and NSST-SR lose the details of the source images in Figures 11 and 12. On the contrary, our method enhanced the contrast and keep more bone structure information.

To summarize the experimental results in accordance with Table 1, the DTCWT and CVT methods performed poor due to low contrast and the data of the objective metrics are lower than other results. The LP method looks unsatisfactory as well, because it did not reserve the information of the MRI image well. The texture and edge are not preserved fully in the fused results of the NSCT-PCNN and SR-CVT.

**Figure 13:**
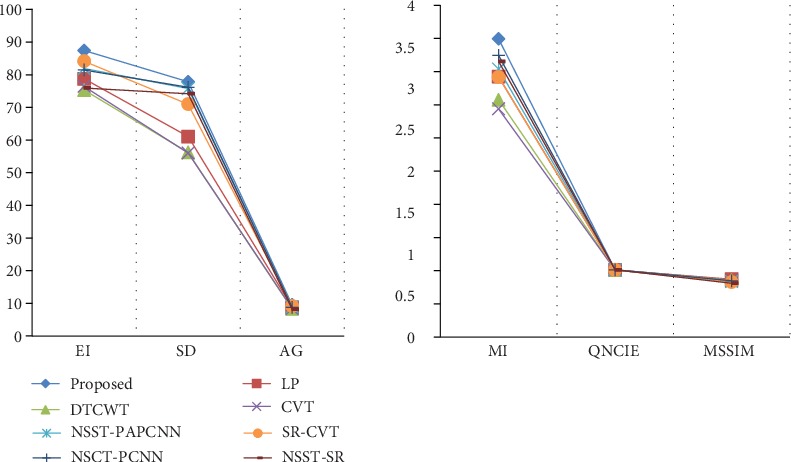
The average objective metrics of the results.

By contrast, the NSST-SR and NSST-PAPCNN achieve clear and high brightness results. But for all this, our performance still makes a bonzer effort, in which energy preservation and detail extraction are to the maximum extent. Among the six metrics, EI, SD, and AG commonly reflect the quality of the result, and the other three metrics including MI, MSSIM, and *Q*_ncie_ make more accurate judgment on image distortion and detail information retention. The higher those metrics above are, the better the quality of the achieved results. As shown in Figures 12 and 13, it can be found from the comprehensive analysis of the numerical values of the objective evaluation metrics of the experiments that our method achieves excellent performance effects on EI, SD, AG, MI, MSSIM, and *Q*_ncie_ metrics, which indicates that the fusion images are significantly better than other contrast methods in terms of contrast, edge detail retention, and image quality.

In addition, when evaluating fusion methods in terms of running time, we make a comparison as shown in Table 2. It is important to note that medical imaging is extremely expensive and the quality of the resulting images should be prioritized during fusion. Since our method directly uses the pretrained CNNs as the feature extractor, we avoid considering the training time of neural network in the time calculation. It is obvious that the running time of the LP method is the fastest than others, and our method spends 6.73 s, which is an acceptable commitment. As previously mentioned, although the LP method runs for a short time, its information retention ability is poor, so are the methods such as DTCWT and CVT. Among the several comparison methods which have obviously achieved excellent fusion effect, the running time of the proposed method is obviously shorter. In a word, compared with the various methods, the proposed method performs better and spends reasonable resources.

### 4.5. Extended Experiment

**Figure 14:**
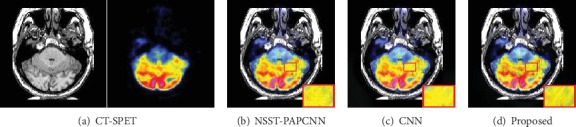
Fusion results of different methods of CT-PET image.

**Figure 15:**
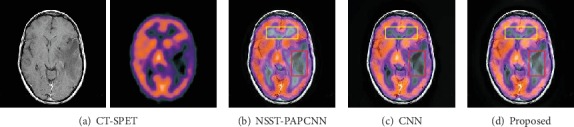
Fusion results of different methods of CT-SPET image.

In order to prove the robustness of our method, we added the experiments to fuse a pair of CT-PET image and CT-SPET image. We also analyzed the performance of the outcome both subjectively and objectively. The results are as shown in Figures 14 and 15.

**Figure 16:**
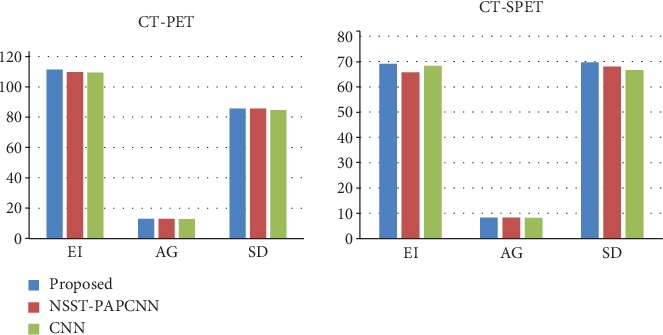
The histogram of each performance metric of fusion results.

We mark the different regions by a red rectangle and enlarge it as shown in Figure 14. Certainly, the result of the proposed method preserves more detail information than the NSST-PAPCNN method and the CNN method which are both well-known fusion strategies and have extreme performance. As shown in Figure 14, the contrast of the red and yellow rectangles in different results is distinctly different. Nonetheless, our method can have a pretty good visual effect in both rectangles. Overall, our method is even ranked at the first place for all the three metrics as shown in Figure 16.

## 5. Conclusions

This paper proposes a brain medical image fusion framework in NSST domain. In this fusion method, the CNN is trained to catch the initial weight from the source images. The NSST is introduced to decompose the source images in the multiscale and direction, and the initial weight is also decomposed by NSST into low- and high-frequency coefficients. The first components of the low-frequency coefficients are fused by an activity level measurement, the low2-frequency made up by the strategy which is designed according to the low-frequency component of the initial weight. The high-frequency coefficients are recombined by the corresponding high-frequency component of the weight. At last, the final result is reconstructed by the inverse NSST. It is proved that our method has excellent performance in both visual effects and objective evaluation by several comparative experiments which consist of different pairs of CT-MR, PET, and SPET images. At the same time, it is indeed proved that the problem is that the weight got out by CNNs' inapplicability on the medical image fusion. Furthermore, we are preparing to do more research about specific medical image and committing to enhance the operational efficiency of the entire integration framework.

## Figures and Tables

**Table 1:** The objective criteria of the results.

	Methods	EI	AG	SD	MI	MSSIM	*Q*_ncie_
Data-1	Proposed	**72.6935**	**7.1552**	**57.9205**	**3.2722**	0.5546	**0.8098**
	LP	38.2196	3.7774	30.0324	2.3271	**0.6351**	0.8054
	DTCWT	34.8032	3.4850	23.2539	1.7309	0.6138	0.8039
	CVT	35.2542	3.4934	23.0665	1.4831	0.5993	0.8033
	NSST-PAPCNN	68.9727	6.6187	56.0234	2.4653	0.5333	0.8060
	SR-CVT	68.6666	6.7490	54.4992	1.9097	0.5147	0.8044
	NSCT-PCNN	66.2118	6.5201	56.4232	2.2337	0.5413	0.8051
	NSST-SR	66.9752	6.6512	53.2715	2.0321	0.5318	0.8047

Data-2	Proposed	**90.0733**	**10.3155**	**69.1393**	4.2751	**0.7368**	0.8123
	LP	83.5461	9.4929	56.4441	3.3646	0.7257	0.8085
	DTCWT	81.8120	9.2944	53.6809	3.1898	0.7261	0.8079
	CVT	82.3516	9.3915	53.6136	3.0625	0.7161	0.8075
	NSST-PAPCNN	85.2049	9.8096	68.2061	3.8848	0.7320	0.8105
	SR-CVT	88.1706	10.0621	68.8248	3.9093	0.7244	0.8107
	NSCT-PCNN	88.2202	9.8461	68.1962	**4.9920**	0.7352	0.8166
	NSST-SR	87.1627	9.6065	68.1307	4.9608	0.7268	**0.8173**

Data-3	Proposed	**74.2671**	**7.7668**	**77.2744**	3.3678	0.7619	0.8094
	LP	72.4568	7.7204	68.6626	**3.4543**	0.7714	**0.8097**
	DTCWT	68.2222	7.1759	65.1873	3.1822	0.7529	0.8083
	CVT	69.9724	7.3461	64.9798	3.1067	0.7326	0.8083
	NSST-PAPCNN	73.0212	7.6503	76.6886	3.3369	0.7662	0.8090
	SR-CVT	67.8324	7.0882	65.8398	3.2342	0.7083	0.8087
	NSCT-PCNN	69.0975	7.3278	75.0149	3.3345	**0.7768**	0.8090
	NSST-SR	63.5011	6.8037	73.9537	3.3862	0.7576	0.8091

Data-4	Proposed	**60.7610**	**6.1253**	**101.008**	**3.3421**	0.6624	**0.8091**
	LP	57.8694	6.0094	72.2220	3.0442	**0.6828**	0.8082
	DTCWT	55.5715	5.6615	67.7378	2.8914	0.6693	0.8078
	CVT	56.0089	5.6900	67.5603	2.8700	0.6629	0.8077
	NSST-PAPCNN	53.0778	5.2554	98.0660	3.1384	0.6078	0.8085
	SR-CVT	59.6227	6.0267	87.1325	3.0472	0.6613	0.8082
	NSCT-PCNN	57.4714	5.7521	99.1359	3.2446	0.6269	0.8087
	NSST-SR	54.7148	5.4708	97.6829	3.1859	0.5902	0.8086

Data-5	Proposed	**145.3350**	16.7255	**86.9107**	**3.5295**	**0.6511**	**0.8089**
	LP	144.0967	**17.0567**	77.7485	3.3466	0.6274	0.8082
	DTCWT	138.1179	16.0188	73.4541	3.1837	0.6284	0.8077
	CVT	140.2991	16.2219	73.5315	3.1072	0.6262	0.8075
	NSST-PAPCNN	143.2695	16.4285	86.2083	3.3442	0.6506	0.8075
	SR-CVT	140.0068	16.1687	75.8551	3.0745	0.6235	0.8074
	NSCT-PCNN	134.0507	15.0210	86.3881	3.2473	0.6473	0.8078
	NSST-SR	123.5401	13.9535	84.4441	3.3016	0.6147	0.8080

Data-6	Proposed	**80.0313**	**8.8360**	**73.7021**	**3.7677**	**0.7539**	**0.8101**
	LP	75.1681	8.5606	60.5508	3.2883	0.7368	0.8084
	DTCWT	72.2479	8.0545	53.0983	2.9707	0.7118	0.8074
	CVT	73.4273	8.1997	52.4865	2.8620	0.6932	0.8071
	NSST-PAPCNN	66.7020	7.4670	68.3819	3.2053	0.7036	0.8079
	SR-CVT	79.4541	8.7675	72.6698	3.6225	0.7352	0.8089
	NSCT-PCNN	72.6950	8.0744	70.6174	3.3051	0.7346	0.8083
	NSST-SR	64.9391	7.3415	66.7485	3.1110	0.6834	0.8076

**Table 2:** The average running times of different methods (times/second).

Methods	LP	DTCWT	CVT	NSST-PAPCNN	SR-CVT	NSCT-PCNN	NSST-SR	Proposed
Time	0.03	0.43	1.98	7.68	2.54	18.73	15.36	6.73

## Data Availability

The data used to support the findings of this study have been deposited in the repository http://www.med.harvard.edu/AANLIB/.
